# Sex Matters: Hippocampal Volume Predicts Individual Differences in Associative Memory in Cognitively Normal Older Women but Not Men

**DOI:** 10.3389/fnhum.2017.00093

**Published:** 2017-03-06

**Authors:** Zhiwei Zheng, Rui Li, Fengqiu Xiao, Rongqiao He, Shouzi Zhang, Juan Li

**Affiliations:** ^1^Center on Aging Psychology, CAS Key Laboratory of Mental Health, Institute of Psychology, Chinese Academy of SciencesBeijing, China; ^2^Department of Psychology, University of Chinese Academy of SciencesBeijing, China; ^3^Magnetic Resonance Imaging Research Center, Institute of Psychology, Chinese Academy of SciencesBeijing, China; ^4^China National Children’s CenterBeijing, China; ^5^State Key Laboratory of Brain and Cognitive Science, Institute of Biophysics, Chinese Academy of SciencesBeijing, China; ^6^Beijing Geriatric HospitalBeijing, China

**Keywords:** associative memory, sex differences, hippocampus, voxel-based morphometry (VBM), elderly

## Abstract

The hippocampus plays a prominent role in associative memory by supporting relational binding and recollection processes. Structural atrophy in the hippocampus is likely to induce associative memory deficits in older adults. Previous studies have primarily focused on average age-related differences in hippocampal structure and memory performance. To date, however, it remains unclear whether individual differences in hippocampal morphometry underlie differential associative memory performance, and whether there are sex differences in the structural correlates of associative memory in healthy older adults. Here, we used voxel-based morphometry (VBM) to examine the extent to which gray matter volume (GMV) of the hippocampus predicts associative memory performance in cognitively normal older adults. Seventy-one participants completed a cued recall paired-associative learning test (PALT), which consists of novel associations and semantically related associations, and underwent magnetic resonance imaging (MRI). We observed worse associative memory performance and larger variability for novel associations than for semantically related associations. The VBM results revealed that higher scores on associative memory for novel associations were related to greater hippocampal GMV across all older adults. When considering men and women separately, the correlation between hippocampal GMV and associative memory performance for novel associations reached significance only in older women. These findings suggest that hippocampal structural volumes may predict individual differences in novel associative memory in older women but not men.

## Introduction

Episodic memory refers to the memory of personally experienced events that occurred at a particular time and place (Tulving, 1985). Normal aging is associated with a decline in episodic memory. The associative deficits hypothesis (ADH) attributes age-related episodic memory decline to difficulties in creating and retrieving associations between single units of information in older adults (Naveh-Benjamin, 2000). As a typical form of episodic memory, associative memory involves the ability to remember inter-item and item-context relationships. Many previous studies have indicated a greater decline in associative memory than item memory in normal aging. This is observed with a wide range of subjects, such as word pairs, picture pairs and face-name pairs (see Old and Naveh-Benjamin, 2008 for a meta-analysis).

It is well established that the hippocampus, which is within the medial temporal lobe (MTL), is especially important in binding and recollecting novel item-item or item-context associations in associative memory (Diana et al., 2007; Hannula and Ranganath, 2009; Blumenfeld et al., 2010; Ranganath, 2010; Westerberg et al., 2012; Eichenbaum, 2017). Functional magnetic resonance imaging (fMRI) studies have documented age-related decreases in hippocampal activation during both relational memory encoding and retrieval (Mitchell et al., 2000; Dennis et al., 2008; Giovanello and Schacter, 2011; Daselaar et al., 2013; Addis et al., 2014). In addition, substantial structural atrophy of the hippocampus may be linked to relational memory impairment in healthy older adults (Resnick et al., 2003; Raz et al., 2004b; Tisserand et al., 2004; Raji et al., 2012; Ta et al., 2012; Fraser et al., 2015). These findings suggest that structural changes in the hippocampus may underlie age-related associative memory deficits.

Previous studies examining the neural correlates of episodic memory in older adults have typically focused on average age-related effects. Recently, neural correlates of individual differences in episodic memory in older adults have attracted considerable attention in cognitive neuroscience studies on aging (Kaup et al., 2011; Nyberg et al., 2012; Salthouse, 2017). Importantly, there is greater individual variability in episodic memory, especially in associative memory, in older adults compared to younger adults (Wilson et al., 2002; Lindenberger and Ghisletta, 2009; Bender et al., 2010; Kilb and Naveh-Benjamin, 2011; Fandakova et al., 2012; Ghisletta et al., 2012; Pudas et al., 2013). Specifically, associative memory decline was observed in some older individuals, but not in others (Fandakova et al., 2015). Given that the hippocampus supports episodic memory by binding item-item or item-context associations, and that greater inter-individual differences in episodic memory have been observed in the elderly, it is plausible that the structure of the hippocampus may be associated with associative memory performance in older adults. However, structural MRI studies of individual differences in associative memory have reported inconsistent results in healthy older adults.

A meta-analysis reported weak positive links between episodic memory and hippocampal volume in older adults (Van Petten, 2004). Furthermore, Rajah et al. (2010) and Bauer et al. (2015) did not find an association between associative memory performance and hippocampal gray matter volume (GMV) in older adults. Interestingly, Becker et al. (2015) found that older adults with better associative memory have larger GMV primarily in the bilateral prefrontal cortex (PFC) rather than the hippocampus. Nevertheless, some structural studies have found positive relationships between hippocampal volumes and scores on associative memory tests in older adults. For example, Shing et al. (2011) reported a positive correlation between hippocampal subfield (CA3/4 and dentate gyrus) volume and associative recognition memory performance. In addition, Zamboni et al. (2013) found a link between hippocampal volume and performance on a visuospatial associative memory task in older adults.

Taken together, previous structural studies investigating associative memory in healthy older adults have reached less consistent conclusions. Some methodological issues may be helpful in explaining why current research has yielded discrepant findings. For example, it has been argued that the cued recall task greatly depends on the hippocampus, and is more likely to recruit the hippocampus than the associative recognition task during associative retrieval (Caplan and Madan, 2016). Consequently, it is more likely to find positive correlations between hippocampal volume and scores on associative memory when a cued recall task is used. In addition, sex might be another important factor to consider. Structural MRI studies have reported sexual dimorphism in the effects of age on brain morphology, and accelerated brain aging in men compared to women was commonly observed (Cowell et al., 1994; Coffey et al., 1998; Resnick et al., 2000; Xu et al., 2000; Good et al., 2001). In particular, previous studies have revealed sex differences in age-related decline in the hippocampus morphology. For example, Raz et al. (2004a) reported a steeper age-related decline in the GMV of the hippocampus in men compared to women. Pruessner et al. (2001) and Li et al. (2014) found that age-related atrophy in the hippocampus was only observed in men. Behaviorally, older women generally perform better than older men on associative memory function (Lamar et al., 2003; Gerstorf et al., 2006; Herlitz and Rehnman, 2008). Thus, it is plausible that sex may be a potential factor resulting in the mixed findings regarding the brain structure correlates of associative memory. Unfortunately, sex differences in the hippocampal structural correlates of individual differences in associative memory have been rarely studied. To date, the only supporting evidence comes from the study of Ystad et al. (2009), which revealed that the left hippocampal volume significantly predicted the free recall scores on verbal learning test for older women but not for men. Given that both free recall and associative memory rely primarily on the recollection process supported by the hippocampus (Yonelinas, 2002), it is plausible to assume that sex differences also exist in the hippocampal structural correlates of associative memory in older adults.

Understanding sex differences in brain structure-behavior associations in normal aging has important implications for appreciating brain-based disorders risk (e.g., mild cognitive impairment, MCI or Alzheimer’s disease, AD), and for early treatment and prevention of these disorders (Mazure and Swendsen, 2016). In the present study, we aimed to use structural MRI to further investigate the sex differences in brain structural basis underlying individual differences in associative memory in normal older adults. We measured participants’ associative memory using the paired-associative learning test (PALT). The PALT is a standardized neuropsychological assessment specifically designed to evaluate associative memory in Chinese (Xu and Wu, 1986; Huo et al., 2014), and has been widely used in previous studies due to its high validity and reliability (Wang et al., 2013; Zheng et al., 2015). The test is designed as a study-test paradigm in which the participants study six novel and six semantically related word pairs and are asked to perform a cued recall test. The study-test procedure repeats three times with different word pair orders each time. Subsequently, the associative memory scores on novel associations and semantically related associations were calculated, respectively. Voxel-based morphometry (VBM) analysis was used to investigate whether local GMV of the hippocampus was associated with scores on the PALT. VBM is a semi-automated whole brain technique for quantifying brain morphological changes (Ashburner and Friston, 2000), and has been widely used to examine the structural brain correlates of cognitive function.

It has been suggested that the cued recall task during associative retrieval is more likely to recruit the hippocampus (Caplan and Madan, 2016), and memory for novel associations between items greatly depends on the hippocampus (Norman and O’Reilly, 2003; Diana et al., 2007; Mitchell and Johnson, 2009; Ranganath, 2010; Eichenbaum, 2017). In addition, age-related decline in the GMV of the hippocampus is relatively modest in older women (Pruessner et al., 2001; Raz et al., 2004a; Li et al., 2014). Relatively preserved GMV in the hippocampus may facilitate associative memory in women but would not influence performance in men who demonstrate a steeper age-related decline in the volume of the hippocampus. As a result, we speculated that there would be sex differences in the correlations between the hippocampal GMV and PALT scores for novel associations, and the GMV of the hippocampus may predict individual differences in associative memory for novel associations in older women but not men. Finally, in order to validate the specificity of the relationship between the GMV of the hippocampus and associative memory, we also performed correlation analyses between the GMV of the clusters obtained in the hippocampal GMV-PALT correlation analyses and scores on neuropsychological tests assessing other cognitive functions (i.e., working memory, semantic memory and executive function).

## Materials and Methods

### Participants

A total of 71 community-dwelling older adults (35 men and 36 women) with normal cognition were recruited from communities near the Institute of Psychology, Chinese Academy of Sciences in Beijing. The inclusion criteria were as follows: (1) age ≥ 60 years; (2) a score ≥ 21 on the Montreal Cognitive Assessment—Beijing Version (MoCA^1^; Yu et al., 2012), (3) right-handedness; (4) no neurological deficits or traumatic brain injury; (5) no dementia or MCI; and (6) preserved activities of daily living (ADL; Lawton and Brody, 1969). The demographic characteristics of the participants are presented in Table 1.

**Table 1:** **Demographic characteristic and neuropsychological measures of the participants (mean and standard deviations)**.

Characteristics	All participants	Men	Women
Age (years)	70.46 (6.04)	71.28 (5.81)	69.67 (6.23)
Education (years)	13.87 (3.26)	14.28 (3.32)	13.47 (3.19)
MoCA	27.00 (2.00)	26.65 (2.11)	27.33 (1.85)
PALT_novel	5.45 (3.55)	4.83 (3.80)	6.05 (3.22)
PALT_related	6.86 (1.31)	6.64 (1.47)	7.07 (1.12)
Digit span forward	7.54 (1.50)	7.69 (1.62)	7.39 (1.39)
Digit span backward	5.02 (1.51)	5.00 (1.67)	5.03 (1.37)
Verbal fluency test	25.04 (6.06)	25.42 (5.33)	24.67 (6.73)
Trail making (seconds)	33.36 (21.71)	32.54 (20.24)	34.10 (23.32)

*Note: Trail making scores were obtained using Trail making B minus Trail making A. MoCA, montreal cognitive assessment. PALT, paired-associative learning test*.

In addition, 54 of the participants (26 men: age, 71.69 ± 5.44 years; education, 15.23 ± 2.72 years; MoCA, 27.08 ± 2.15, and 28 women: age, 70.86 ± 6.54 years; education, 13.89 ± 3.20 years; MoCA, 27.46 ± 2.01) completed the digit span forward (DSF) and digit span backward (DSB) tests (Wechsler, 1981), verbal fluency test (VFT, Spreen and Strauss, 1998) and trail making tests A and B (TMT, Reitan, 1992).

The study protocol was approved by the Ethics Committee of the Institute of Psychology, Chinese Academy of Sciences, and written informed consent was obtained from all participants.

### Neuropsychological Measures

The PALT (Xu and Wu, 1986) was used to assess participants’ associative memory performance. For this test, the participants first studied 12 word pairs consisting of nouns with six novel associations (e.g., teacher-railway) and six semantically related associations (e.g., sun-moon). During the study, all 12 word pairs were read at a rate of 1 s per word pair, with intervals of 2 s between two pairs. After the study session, the participants were asked to complete a cued recall task in which the first word of the pair was provided and they had to recall the other paired word within 5 s of hearing the first word. A correctly recalled word was scored 0.5 for the semantically related associations and 1 for the novel associations. The procedure was repeated three times with different word pair orders each time. The scores on novel associations (range: 0–18) and semantically related associations (range: 0–9) were calculated, respectively.

In addition, MoCA, DSF and DSB tests, category VFT and TMT A and B were used to assess global cognition, working memory, semantic memory and executive function, respectively.

### MR Image Acquisition

A 3-Tesla Siemens Trio scanner (Erlangen, Germany) equipped for echo planar imaging (EPI) at the Beijing MRI Center for Brain Research was used for image acquisition. A high-resolution, 3-D T1-weighted structural image was acquired for each participant, using a magnetization-prepared rapid gradient echo (MPRAGE) sequence: 176 slices, acquisition matrix = 256 × 256, voxel size = 1 × 1 × 1 mm^3^, TR = 1900 ms, TE = 2.2 ms and flip angle = 9°. T1-weighted structural MRI images were used for VBM analysis.

## Data Analysis

### Image Processing

VBM was performed using the Statistical Parametric Mapping program (SPM8^2^, Wellcome Trust Center for Neuroimaging, London, UK), and the toolbox for Data Processing and Analysis of Brain Imaging (DPABI V2.1^3^; Yan et al., 2016) in MATLAB R2012b (Mathworks, Inc., Natick, MA, USA). Before processing, all of the structural images were visually inspected for artifacts and the origin of the images was manually set at the anterior commissure for each participant. All 71 participants were included in the VBM analysis.

The structural MR images were first normalized and segmented into gray matter, white matter and cerebrospinal fluid (Ashburner and Friston, 2005). Following segmentation, the gray matter images were processed to create study-specific gray matter population templates using the DARTEL algorithm. After an initial affine registration of the gray matter DARTEL template to the tissue probability map in the Montreal Neurological Institute (MNI) space, we applied a non-linear warping of the gray matter images to the DARTEL gray matter template in the MNI space and resampled to 1.5 × 1.5 × 1.5 mm^3^. The gray matter values of each voxel were modulated by multiplying the gray matter concentration map by the nonlinear determinants derived during spatial normalization. Finally, the modulated gray matter images were smoothed using an 8-mm full-width-at-half-maximum isotropic Gaussian kernel. The images were then used for the following statistical analysis.

### Statistical Analyses

The processed gray matter images were entered into a regression model using DPABI to measure correlations between regional GMV and scores on neuropsychological measures.

**Figure 1:**
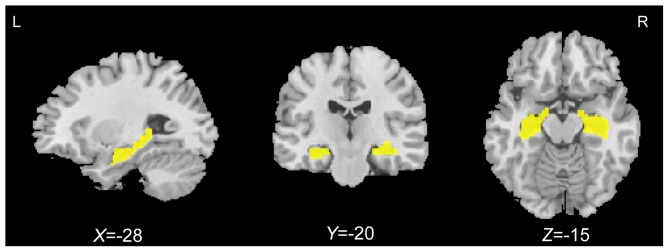
**Bilateral hippocampal regions of interest (yellow) defined using the automated anatomical labeling (AAL) atlas**.

A hypothesis-driven analysis in which the hippocampal GMV was correlated with associative memory performance for all older adults was performed first. Before the statistical analysis, the Wake Forest University Pick Atlas (Maldjian et al., 2003) was used to define *a priori* region of interest (ROI) including the bilateral hippocampi according to the automated anatomical labeling (AAL) atlas. Figure 1 shows the slice view of the ROI applied to each of the participants. Correlation analyses between the GMV and PALT scores on novel associations and semantically related associations within the predefined ROI were performed separately. A cluster-based multiple comparisons correction of *p* < 0.005 (individual voxel *p* < 0.001) was performed with Monte Carlo simulation in AlphaSim (Forman et al., 1995; Huang et al., 2016; Zhou et al., 2016). Accordingly, clusters were considered significant at a cluster size of > 91 voxels (307 mm^3^) for correlation analyses within the ROI mask (4473 voxels).

We also conducted additional GMV-PALT correlation analyses using a whole-brain approach to validate the results of the ROI analysis. The statistical threshold was set at *p* < 0.05 using the AlphaSim correction (individual voxel *p* < 0.001) and a minimum cluster size of 1015 voxels (3425 mm^3^) within a whole brain gray matter mask (540,194 voxels).

All of the correlation analyses were first performed across all older adults. To regress any confounding effects of age, gender, education level and global cognitive performance (i.e., MoCA), we entered these variables as covariates into the regression model. We then conducted additional within sub-group GMV-behavior correlation analyses separately for older men and women with age, education level and MoCA as covariates. Between-group comparisons were conducted with Fisher’s *r* to *Z* transformation to directly compare the sex differences in GMV-behavior correlation.

Finally, in order to validate the specificity of the relationship between the GMV of the clusters observed in the ROI analyses and the performance on PALT, we performed correlation analyses between the GMV of the clusters obtained in the ROI correlation analyses and other neuropsychological measures (i.e., DSF test, DSB test, category VFT and TMT) to validate the specific role the hippocampus plays in associative memory in older adults.

## Results

### Behavioral Results

**Figure 2:**
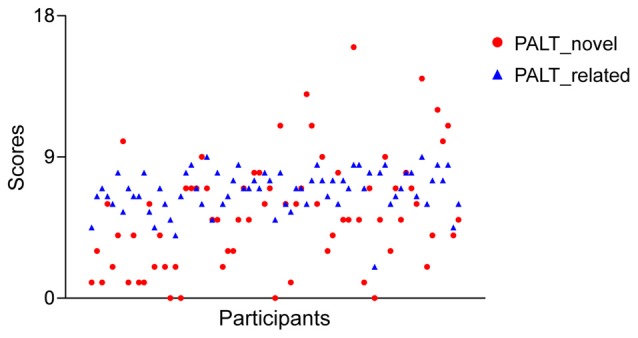
**A scatter plot of the associative memory performance across all older adults for each measure of the paired-associative learning test (PALT)**.

Table 1 displays the demographic characteristics and neuropsychological measures of the participants. A paired samples *t*-test for the associative learning test across all participants revealed that older adults had worse performance for the novel associations compared to the semantically related associations (5.45 vs. 6.86; *t* = −3.84, *p* < 0.001). In addition, generally reduced inter-individual variance was observed for the semantically related associations compared to the novel condition (*SD*: 1.31 vs. 3.55; Figure 2). An independent samples *t*-test revealed no significant sex differences in demographic characteristics or neuropsychological performance between older men and women.

### Correlation Analyses between ROI GMV and PALT

All 71 participants (35 men and 36 women) were included in the correlation analyses between ROI GMV and PALT.

### Novel Associations

**Figure 3:**
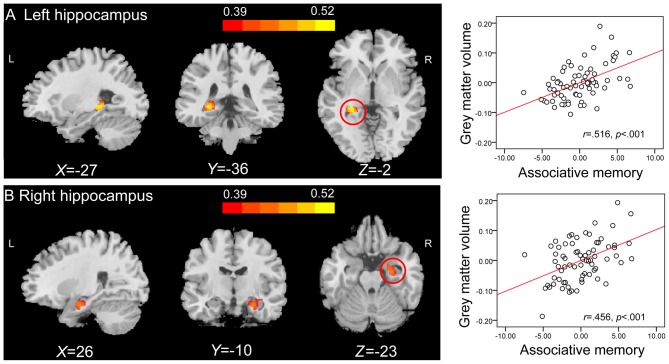
**Region of interest (ROI) correlation analyses between gray matter volume (GMV) and scores on associative memory for novel associations.** The left panel shows that GMV of the left hippocampus (peak Montreal Neurological Institute (MNI) coordinate: −27, −36, −2; number of voxels: 334) **(A)** and the right hippocampus (peak MNI coordinate: 26, −10, −23; number of voxels: 425) **(B)** are significantly correlated with scores on associative memory for novel associations across all older adults. Bars at the top show the correlation values. The right panel shows the partial regression plots between the GMV of the peak coordinate in the left hippocampus **(A)** and the right hippocampus **(B)** and scores on associative memory for novel associations after controlling age, gender, education level and montreal cognitive assessment (MoCA).

**Figure 4:**
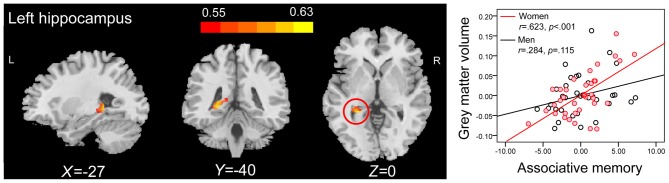
**ROI correlation analyses between GMV and scores on associative memory for novel associations in older women.** The left panel shows that GMV of the left hippocampus (peak MNI coordinate: −27, −40, 0; number of voxels: 309) is significantly correlated with scores on associative memory for novel associations in older women. Bars at the top show the correlation values. The right panel shows the partial regression plots between the GMV of peak coordinate in the left hippocampus and scores on associative memory for novel associations in older men and women after controlling age, education level and MoCA.

Correlation analysis revealed that the GMVs of both the left hippocampus (peak MNI coordinate: −27, −36, −2; number of voxels: 334; *r* = 0.516; Figure 3A), and the right hippocampus (peak MNI coordinate^4^: 26, −10, −23; number of voxels: 425; *r* = 0.456; Figure 3B) were positively related to PALT scores on novel associations across all older adults. When considering men and women separately, older women had a positive correlation between the left hippocampal GMV (peak MNI coordinate: −27, −40, 0; number of voxels: 309; *r* = 0.623; Figure 4) and performance on PALT for novel associations. There were no significant correlations in older men. We extracted the GMV of peak coordinate in the left hippocampus and performed a partial correlation analysis between GMV and PALT scores for novel associations separately for older men and women with age, education level and MoCA as covariates. A further analysis directly comparing the correlations between the two groups revealed greater correlation coefficient for older women than for older men (Fisher’s *r* to *Z* = 1.765, *p* = 0.038, one-tail test).

### Semantically Related Associations

There were no significant correlations between the ROI GMV and PALT scores on semantically related associations regardless of whether the analyses were performed across all older adults or separately for men and women.

### Whole-Brain Correlation Analyses between GMV and PALT

All 71 participants (35 men and 36 women) were included in the whole-brain correlation analyses between GMV and PALT.

### Novel Associations

**Figure 5:**
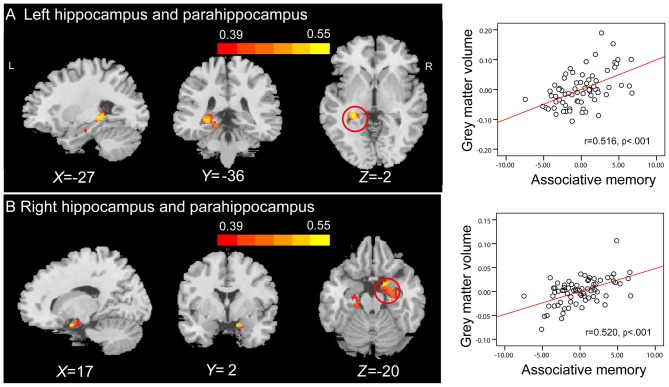
**Whole-brain correlation analyses between GMV and scores on associative memory for novel associations.** The left panel shows that GMV in anatomical clusters that mainly include the left hippocampus and parahippocampal gyrus (peak MNI coordinate: −27, −36, −2; number of voxels: 1441) **(A)** and the right hippocampus and parahippocampal gyrus (peak MNI coordinate: 17, 2, −20; number of voxels: 2033) **(B)** are significantly correlated with scores on associative memory for novel associations in older adults. Bars at the top show the correlation values. The right panel shows the partial regression plots between the GMV of the peak coordinate in anatomical clusters that mainly include the left hippocampus and parahippocampal gyrus **(A)** and the right hippocampus and parahippocampal gyrus **(B)** and scores on associative memory for novel associations after controlling age, gender, education level and MoCA.

**Figure 6:**
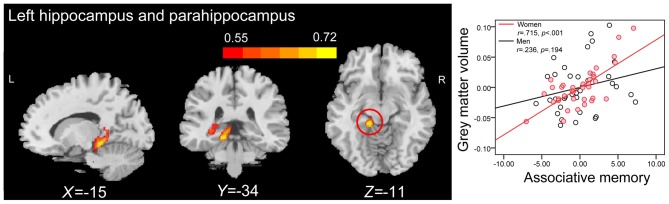
**Whole-brain correlation analyses between GMV and scores on associative memory for novel associations in older women.** The left panel shows that GMV in anatomical cluster that mainly include the left hippocampus and parahippocampal gyrus (peak MNI coordinate: −15, −34, −11; number of voxels: 2393) is significantly correlated with scores on associative memory for novel associations in older women. The right panel shows the partial regression plots between the GMV of the peak coordinate in the anatomical cluster that mainly include the left hippocampus and parahippocampal gyrus and scores on associative memory for novel associations in men and women after controlling age, education level and MoCA.

Whole-brain correlation analysis only revealed significant positive correlations between the GMVs in anatomical clusters that mainly included the left hippocampus and parahippocampal gyrus (peak MNI coordinate: −27, −36, −2; number of voxels: 1441; *r* = 0.516; Figure 5A), and the right hippocampus and parahippocampal gyrus (peak MNI coordinate: 17, 2, −20; number of voxels: 2033; *r* = 0.520; Figure 5B) and PALT scores on novel associations across all older adults. Considering men and women separately, older women had a positive correlation between the GMV in an anatomical cluster that mainly included the left hippocampus and parahippocampal gyrus (peak MNI coordinate: −15, −34, −11; number of voxels: 2393; *r* = 0.715; Figure 6) and performance on PALT for novel associations. There were no significant associations in older men. We extracted the GMV of peak coordinate in the cluster that mainly include the left hippocampus and parahippocampal gyrus and performed a partial correlation analysis between GMV and PALT scores for novel associations separately for older men and women with age, education level and MoCA as covariates. Subsequent analysis directly comparing the correlations between the two groups revealed greater correlation coefficient for older women than for older men (Fisher’s *r* to *Z* = 2.647, *p* = 0.004, one-tail test).

### Semantically Related Associations

Correlation analysis between whole-brain GMV and PALT scores on semantically related associations revealed no significant associations, regardless of whether the analyses were performed across all older adults or separately for men and women.

In summary, both ROI and whole-brain correlation analyses revealed that bilateral hippocampal volumes were significantly associated with scores on the PALT for novel associations across all older adults. No significant correlations were found for the semantically related associations. When considering men and women separately, only women displayed highly significant and positive correlations between left hippocampal GMV and PALT scores on novel associations.

### Correlation Analyses between GMV and Other Neuropsychological Measures

Fifty-four participants (26 men and 28 women) were included in the correlation analyses between GMV and other neuropsychological measures.

We extracted the GMV of peak coordinate of the clusters observed in the ROI correlation analyses between GMV and PALT for novel associations (i.e., left and right hippocampus), and separately performed correlation analyses between GMV and scores on DSF test, DSB test, category VFT and TMT across all older adults with age, gender, education level and MoCA as covariates. The results revealed no significant correlations between the GMV of the left hippocampus and performance on DSF (*r*_(48)_ = 0.071, *p* = 0.624), DSB (*r*_(48)_ = 0.204, *p* = 0.155), VFT (*r*_(48)_ = −0.107, *p* = 0.460), or TMT (*r*_(48)_ = −0.144, *p* = 0.317) at a Bonferroni correction threshold of 0.0125 (0.05/4). In the same conditions, there were also no significant correlations between the GMV of the right hippocampus and performance on DSF (*r*_(48)_ = −0.155, *p* = 0.283), DSB (*r*_(48)_ = 0.037, *p* = 0.801), VFT (*r*_(48)_ = −0.173, *p* = 0.230), or TMT (*r*_(48)_ = −0.009, *p* = 0.952).

We also extracted the GMV of peak coordinate of the cluster observed in the ROI correlation analyses between GMV and PALT for novel associations in older women. We then separately performed correlation analyses between GMV and scores on the above neuropsychological measures for older women with age, education level and MoCA as covariates. There were no significant correlations between the GMV of the left hippocampus and performance on DSF (*r*_(23)_ = −0.151, *p* = 0.471), DSB (*r*_(23)_ = 0.054, *p* = 0.797), VFT (*r*_(23)_ = −0.220, *p* = 0.291), or TMT (*r*_(23)_ = −0.260, *p* = 0.209) at a Bonferroni correction threshold of 0.0125 (0.05/4).

These analyses suggest that the hippocampus might be functionally specific for associative memory.

## Discussion

Using VBM analysis, we investigated the structural brain correlates of individual differences in associative memory in healthy older adults. The results revealed that GMVs in bilateral hippocampi were positively correlated with associative memory performance for novel associations across all older adults. However, when considering men and women separately, the relationship between hippocampal volume and novel associative memory performance reached significance only in older women. These findings highlight the important role of the hippocampus in remembering novel associative representations in older women.

A number of studies provide evidence documenting morphologic sex differences in adult brain structure (Cosgrove et al., 2007; Sacher et al., 2013; Ruigrok et al., 2014; Gur and Gur, 2017). It has been reported that women have a higher percentage of gray matter (Gur et al., 1999; Leonard et al., 2008), greater cortical thickness (Im et al., 2006; Luders et al., 2006; Sowell et al., 2007) and higher corpus callosum morphology (Mitchell et al., 2003) than men. In addition, accelerated brain aging in men compared to women have been observed (Cowell et al., 1994; Coffey et al., 1998; Resnick et al., 2000; Xu et al., 2000; Good et al., 2001). In particular, structural MRI studies in humans have revealed steeper age-related decline in hippocampus morphology in men compared to women (Pruessner et al., 2001; Raz et al., 2004a; Li et al., 2014). Given that the hippocampus is especially critical for novel associative memory encoding and retrieval (Diana et al., 2007; Ranganath, 2010; Eichenbaum, 2017), we speculated that the relatively preserved hippocampus GMV would play a more important role in associative memory for novel associations in older women compared with older men, and thus structural volumes of the hippocampus are more likely to be associated with individual differences in associative memory for novel associations in older women but not men. Our results confirmed this speculation by demonstrating that structural volume of the hippocampus could account for inter-individual variations in novel associative memory only in older women.

The correlation analyses revealed significant positive associations between the GMV of the hippocampus and novel associative memory in older women, indicating that larger GMV of the hippocampus was associated with better novel associative performance. Since the hippocampus is believed to support associative memory by supporting the binding and recollection of novel relational associations (Diana et al., 2007; Ranganath, 2010; Eichenbaum, 2017), positive correlation for older women suggests that greater GMV of the hippocampus facilitates memory performance when binding and recollection are required during associative memory encoding and retrieval. The present findings are actually quite consistent with Ystad et al. (2009)’s observations that the hippocampal volume significantly predicted the free recall performance for older women but not for men, given that both novel associative memory and free recall rely primarily on the recollection process supported by the hippocampus (Yonelinas, 2002). It is reasonable to expect that the regional hippocampal GMV may be an indicator of age-related changes in associative memory function, and may even be a potential biomarker for the early detection of the deterioration of associative memory in cognitively normal older women.

The absence of significant associations in older men may imply that older men are more impaired in binding and/or recollection processes because of their steeper age-related atrophy in the hippocampus (Pruessner et al., 2001; Raz et al., 2004a; Li et al., 2014), and thus the extent of GMV in hippocampus does not affect their associative memory performance. Another potential reason why older men did not show significant correlation may be that their novel associative memory is related to strategic processes rather than the binding or recollection process, given that both associative and strategic components may contribute to associative memory performance (Shing et al., 2010). If that were the case, we would speculate that the GMV of PFC, due to its important role in elaborative organization and strategic retrieval (Simons and Spiers, 2003; Mitchell and Johnson, 2009), would have been related to associative memory performance in older men. Unfortunately, in the whole brain correlation analyses between GMV and PALT, no significant correlations with PALT scores on novel associations in the PFC were found in older men, which did not support this assumption. In addition, although many have suggested the hormonal and genetic influences on sex differences in brain and behavior (Lentini et al., 2013; Kight and McCarthy, 2017), the neurobiological basis for the sex differences in the relationships between hippocampal structure and associative memory remains unknown.

Contradictorily, some previous investigations of the brain structure correlates of episodic memory did not identify positive relationships between the hippocampal volumes and associative memory for novel associations in older adults (Rajah et al., 2010; Bauer et al., 2015; Becker et al., 2015). The inconsistent findings may be associated with methodological differences between studies, such as variations in test formats, volumetric procedures, and analytical strategies (Kaup et al., 2011; Ezzati et al., 2016). For example, in the present study, we used a cued-recall paired-associative learning task, which was more likely to recruit the hippocampus than an associative recognition task used in Becker et al. (2015), to measure older individuals’ associative memory performance (Caplan and Madan, 2016). We speculate that the cued-recall PALT is a more hippocampus-dependent task, and is more likely to drive the present significant correlations between hippocampal volumes and scores on the PALT. In addition, we defined the ROI of hippocampus based on automated procedures, while Rajah et al. (2010) defined the ROI by manually tracing the brain region. Most importantly, previous studies exploring the structural neural correlates of associative memory usually performed the correlation analyses across the whole group of participants (e.g., Rajah et al., 2010; Bauer et al., 2015; Becker et al., 2015), whereas we paid more attention to sex differences in the correlation patterns between GMV and associative memory performance. These may have contributed to the inconsistent findings of previous studies.

In whole brain correlation analyses, the GMV of the parahippocampal gyrus also had significant correlations with the PALT scores for novel associations only in older women, indicating that larger GMV of the parahippocampal gyrus was associated with better novel associative performance in older women but not men. Neuroimaging studies have demonstrated that the parahippocampal gyrus within the MTL plays a critical role in encoding and recollecting detailed contextual information (Kirwan and Stark, 2004; Diana et al., 2007, 2009; Ranganath, 2010). In addition, the parahippocampal gyrus is also a region within the MTL that is vulnerable to aging effects (Resnick et al., 2003; Tisserand et al., 2004; Raz et al., 2005; Raji et al., 2012). Unfortunately, less attention has been paid to sexual dimorphism in age-related structural atrophy in the parahippocampal gyrus. Nevertheless, the present findings of sex differences in associations between the GMV of the parahippocampal gyrus and novel associative memory may suggest that greater GMV of the parahippocampal gyrus may facilitate memory performance by supporting the encoding and retrieval of contextual information in older women but men. Our findings provide new evidence for the importance of the parahippocampal gyrus in associative memory and suggest that the volumes of the parahippocampal gyrus may be relevant to the individual differences in associative memory in healthy older women.

Interestingly, both ROI and whole-brain correlation analyses revealed that there were no significant correlations between the hippocampal, and parahippocampal GMV and the PALT scores on semantically related associations in older adults. First, it is well established that both the hippocampus and parahippocampus play critical roles in remembering novel relational associations rather than related associations (Diana et al., 2007; Ranganath, 2010; Eichenbaum, 2017). Second, in line with previous studies indicating that older adults perform better when the episodic components used are already associated in memory (Naveh-Benjamin, 2000; Naveh-Benjamin et al., 2003), our behavioral results indicated better performance on semantically related associations in older adults. Critically, older adults also had smaller inter-individual variability for semantically related associations compared to novel associations. Finally, as can be seen from the Figure 2, the data suggests that there was a ceiling effect in memory performance on semantically related condition due to its low-difficulty level. These observations may explain why the correlations between hippocampal volume and scores on the PALT were not statistically significant for semantically related associations.

It is well known that the PFC is important for associative memory, as it implements executive control processes, such as elaborative and organized operations (Simons and Spiers, 2003; Mitchell and Johnson, 2009). It has been shown that there are great age-related gray matter reductions in the PFC (Raz et al., 2005; Fjell et al., 2009; Di et al., 2014), particularly in the inferior frontal subregions (Resnick et al., 2003). This atrophy may result in age-related memory deficits by affecting the use of self-initiated elaborative memory strategies in older adults (Craik and Rose, 2012; Kirchhoff et al., 2014). In a previous structural study of associative memory, Becker et al. (2015) found that older adults with larger GMVs in the bilateral PFC had better associative memory. This underscores the contribution of GMV of the PFC to individual differences in associative memory in older adults. Interestingly, in the present study, no clusters other than the hippocampus and parahippocampus had significant correlations with PALT scores in older women in whole brain correlation analyses. Again, methodological differences (e.g., test formats or analytical strategies) between studies may give rise to different patterns across studies.

Some limitations should be noted. First, a relatively small sample of older adults was included in the present study. The sex differences found in the correlation analyses within subgroups and the validation analyses of functional specificity of the hippocampus for associative memory should be treated with caution. A larger sample size should be used to assess the structural correlates of inter-individual differences in associative memory in older adults in future studies. Second, we only recruited cognitively healthy older adults (age ≥ 60 years) in the present study. It should be noted that aging is a process that represents any changes (including physical, psychological and social change) in a human being over time (Bowen and Atwood, 2004). It is necessary to explore the aging effects on hippocampal structural correlates of associative memory across longer adulthood in future studies. In addition, longitudinal studies are required to examine the developmental trajectory of the relationship between hippocampal structure and associative memory. Finally, it has been suggested that hippocampal volume is especially sensitive to pathology-related atrophy and individual variation in patients with MCI or those with AD (Hua et al., 2008). Positive correlations between hippocampal volume and memory performance are especially apparent in samples of these patients (Van Petten, 2004). Including individuals with MCI and AD, may to some extent, provide clinical clues regarding the changing trajectory of associative memory during aging.

## Conclusion

We observed that GMVs in the hippocampus may predict associative memory ability in cognitively normal older women but men. This provides new evidence for sexual dimorphism in the importance of the hippocampus in associative memory. Hippocampal volume may be an indicator of associative memory processing in aging, or a potential biomarker for the early detection of associative memory deterioration, especially in older women. Examining sex differences in future studies of the cognitive neuroscience of aging may help us to understand the aging brain better.

## Author Contributions

ZZ conceived the idea, analyzed and interpreted the data and drafted part of the manuscript. RL, FX, RH and SZ assisted the analysis and interpretation of data. JL conceived the idea, and participated in the writing and revision of the manuscript.

## Funding

This research was supported by the National Natural Science Foundation of China (31271108, 31470998, 31671157), the National Science and Technology Pillar Program of China (2009BAI77B03), the Pioneer Initiative of the Chinese Academy of Sciences, Feature Institutes Program (TSS-2015-06), the Scientific Foundation of Institute of Psychology, Chinese Academy of Sciences (Y5CX131005) and CAS Key Laboratory of Mental Health, Institute of Psychology, Chinese Academy of Sciences (KLMH2015ZG06).

## Conflict of Interest Statement

The authors declare that the research was conducted in the absence of any commercial or financial relationships that could be construed as a potential conflict of interest.

## Footnotes

^1^The MoCA is a cognitive screening tool for mild cognitive impairment (MCI) and mild dementia, and can be used to assess global cognitive function.

^2^http://www.fil.ion.ucl.ac.uk/spm

^3^http://rfmri.org/dpabi

^4^The peak MNI coordinate indicates the coordinate that has greatest correlation with the associative memory performance.
